# Cr (VI) and Pb (II) Removal Using Crosslinking Magnetite-Carboxymethyl Cellulose-Chitosan Hydrogel Beads

**DOI:** 10.3390/gels9080612

**Published:** 2023-07-28

**Authors:** Nur Maisarah Mohamad Sarbani, Endar Hidayat, Kanako Naito, Yoshiharu Mitoma, Hiroyuki Harada

**Affiliations:** 1 Graduate School of Comprehensive and Scientific Research, Prefectural University of Hiroshima, Shobara 727-0023, Japan; nur1maisarah@gmail.com (N.M.M.S.); hidayatendar1@gmail.com (E.H.); naito@pu-hiroshima.ac.jp (K.N.); mitomay@pu-hiroshima.ac.jp (Y.M.); 2 Department of Life and Environmental Science, Faculty of Bioresources Science, Prefectural University of Hiroshima, Shobara 727-0023, Japan

**Keywords:** carboxymethyl–cellulose, chitosan, Pb (II) removal, Cr (VI) removal, adsorption, adsorption capacity, desorption

## Abstract

Heavy metals, such as chromium (VI) and lead (II), are the most common pollutants found in wastewater. To solve these problems, this research was intended to synthesize magnetite hydrogel beads (CMC-CS-Fe_3_O_4_) by crosslinking carboxymethyl cellulose (CMC) and chitosan (CS) and impregnating them with iron oxide (Fe_3_O_4_) as a potential adsorbent to remove Cr (VI) and Pb (II) from water. CMC-CS-Fe_3_O_4_ was characterized by pH_zpc_, scanning electron microscopy (SEM), and Fourier-transform infrared spectroscopy (FTIR). Batch removal experiments with different variables (CMC:CS ratio, pH, initial metals concentration, and contact time) were conducted, and the results revealed that CMC-CS-Fe_3_O_4_ with a CMC:CS (3:1) ratio had the best adsorption capacity for Cr (VI) and Pb (II) at pH levels of 2 and 4, respectively. The findings of this research revealed that the maximum adsorption capacity for Cr (VI) and Pb (II) were 3.5 mg/g and 18.26 mg/g, respectively, within 28 h at 30 ℃. The adsorption isotherm and adsorption kinetics suggested that removal of Cr (VI) and Pb (II) were fitted to Langmuir and pseudo-second orders. The highest desorption percentages for Cr (VI) and Pb (II) were 70.43% and 83.85%, achieved using 0.3 M NaOH and 0.01 M N·a2EDTA, respectively. Interestingly, after the first cycle of the adsorption–desorption process, the hydrogel showed a sudden increase in adsorption capacity for Cr (VI) and Pb (II) until it reached 7.7 mg/g and 33.0 mg/g, respectively. This outcome may have certain causes, such as entrapped metal ions providing easy access to the available sites inside the hydrogel or thinning of the outer layer of the beads leading to greater exposure toward active sites. Hence, CMC-CS-Fe_3_O_4_ hydrogel beads may have potential application in Cr (VI) and Pb (II) removal from aqueous solutions for sustainable environments.

## 1. Introduction

Heavy metals are among the most common pollutants found in wastewater, which is released from various sources, such as the metal refining, batteries, mining, and agricultural industries. Chromium (Cr) and Lead (Pb) are kinds of toxic heavy metals frequently found in wastewater. Without proper removal treatment of these heavy metals from contaminated wastewater, it might lead to their accumulation and build up in wastewater because they are difficult to degenerate [1] and can generate cumulative loads in water and organisms due to their bio-accumulative properties [2,3]. Chromium tends to diffuse into suspended particles in wastewater, leading to build-up in the sediment, thereby remaining in wastewater for a long time. Moreover, continuous exposure to these contaminants will degrade the quality of water and soil and therefore be extremely harmful to aquatic organisms, humans, and the environment due to their toxicity and non-biodegradability [4]. According to the World Health Organization (WHO), the parameter limit for chromium (VI) in wastewater is 0.1 ppm [5], whereas for Pb (II), it is 0.01 ppm for wastewater and 0.1 ppm for soil [6].

Safe and clean water is necessary to protect the public from health risks. Since heavy metals are non-biodegradable, carcinogenic, and mutagenic to living organisms [7], an effective solution is needed to reduce and remove these pollutants in water. To reduce the threats to the environments, various methods have been applied to remove metals from wastewater. Adsorption is a commonly used technique for heavy metal removal due to its simplicity, flexibility, and effectivity [8]. Adsorption is the adhesion of atoms, ions, or molecules from a gas, liquid, or dissolved solid onto a solid surface by physical forces or weak chemical bonds [9,10]. This process creates a film of the adsorbate on the surface of the adsorbent. Currently, researchers have more interest in natural adsorbent materials made from non-toxic materials, such as natural polymers and biomass, which have high effectivity, porous structures, and low cost.

There are a wide range of natural and low-cost adsorbents that have been discovered for heavy metal removal, such as Cr (VI), Pb (II), and many others. These natural adsorbents include biomass wastes, such as rice husks, coffee husks, orange peels, corn husks, and non-agricultural biomass, such as chitin, clay mineral, zeolite, etc. [11,12,13]. The usage of rice husks with incorporation of iron oxide is able to effectively remove Cr (VI) with adsorption capacity of 63.69 mg/g [14]. Moreover, a study conducted by Kobayashi et al. [15] reported that zeolites have high porosity and yielded maximum adsorption of 30.7 mg/g for Pb (II). Moreover, the cost for adsorption processes using natural adsorbents is relatively low compared to commercial adsorbents. A previous study conducted by Almeida et al. [16] reported that bentonite calcined clay was used to remove copper with maximum adsorption capacity of 19.06 mg/g. Clay is commonly used as an adsorbent due to its effective cost, being 20 times less expensive than activated carbon.

In recent years, there has been increasing interest in hydrophilic structures, such as hydrogels. Hydrogels are considered as among the reliable forms of adsorbents due to their water absorbency and retention properties. Furthermore, this form allows for the formation of complex structures with pollutants due to the presence of functional groups within the structural network of the hydrogel. These hydrogels, which are typically produced from chitosan, cellulose, alginate, starch, and their derivatives, have many advantages because of their biodegradability, non-toxicity, reusability, and renewability [17,18].

Chitosan (CS) is a deacylated derivative of chitin, and it is a fibrous compound normally derived from crustaceans and shellfish, such as crabs, shrimp, and others [19]. Chitosan is a polysaccharide that has linear chain composed of 1,4-linked residues of glucosamine and N-acetyl-glucosamine residues [20]. Recently, in 2019, the market value of chitosan was growing as a result of the increasing application of chitosan as biopolymer in the water-treatment, pharmaceutical, and food industries [21]. This polymer is composed of reactive functional groups, known as primary amino groups and hydroxyl groups, which are available for modification to enhance the properties or introduce new features to this polymer. Abundant numbers of amino groups in chitosan are responsible for its solubility in acidic solutions, such as acetic acid solution [22]. Thus, the structure and functionality of chitosan are highly dependent on pH conditions. The biodegradability and biocompatibility, as well as functional amino groups, of this polymer have contributed to the physicochemical and cationic properties of chitosan being widely used in many industrial applications.

Carboxymethyl cellulose (CMC) is basically derived from cellulose with carboxymethyl (–CH_2_COOH) bound to it. CMC has high solubility, viscosity, and adsorptive functionality due to the presence of active functional groups, such as hydroxyl (–OH), carboxyl (–COO), and carbonyl groups (–C=O) [23,24]. CMC is one of the most common natural polymers containing biodegradable material, and it has high durability since it has great resistance to hydrolysis. Additionally, the presence of –OH in cellulose allows it to undergo various modifications, such as esterification, oxidation, and cross-linking reactions, to create the desired functionalities of CMC. Moreover, the high porosity and large surface area of CMC nanocomposites or films have been found to have good functionality in metal adsorption. A previous study by Olad et al. [25] revealed that porous and high-surface area material produced from the ion crosslinking of CMC could significantly enhance the adsorption capacity of Cr (VI) in wastewater.

Therefore, the concept of ionic interactions between carboxymethyl cellulose (CMC) and chitosan (CS) polymers to form hydrogels might be a good approach to implement for adsorption principles to study its functions and potential to remove Cr (VI) and Pb (II). According to Zhang et al. [26], the binding of chitosan to cellulose has been proven to greatly increase and enhance the structural properties, integrity, flexibility, and elasticity of hydrogels. CMC-chitosan composites have been formed through ionic crosslinking of CMC and chitosan due to the inter-charge and intra-charge between anionic and cationic polymers of CMC and chitosan, correspondingly [27,28]. Moreover, Sharif et al. [29] reported that a composite from chitosan and CMC was capable of removing around 50–90% of heavy metals [Cr (VI), Ni (II), Pb (II), Cu (II), Zn (II)] from aqueous solution.

In this study, we describe the potential of crosslinking CMC, chitosan, and magnetic components to produce novel adsorbent magnetic hydrogel beads. Previous researchers have published works related to the use of chitosan or carboxymethyl cellulose as pollutant adsorbents, drug carriers, or hydrogels in various industries; however, there is no information regarding the use of crosslinking CMC–chitosan hydrogels with magnetite properties as a metal adsorbent. Other than facilitating the separation process, magnetite iron oxide (Fe_3_O_4_) also has an additional function of adsorbing contaminants. It has been commonly used in wastewater treatment owing to its advantages, such as low cost, adjustability to a wide range of pH values, efficiency, and able to regenerate [30,31]. Fe_3_O_4_ nanocomposite is known to have a large specific surface area, to yield a high adsorption capacity [32], and to increase the stability of adsorbents by providing a large number of active sites on the surface of adsorbents [33].

Therefore, this research aimed to synthesize magnetite hydrogel beads (CMC-CS-Fe_3_O_4_) by crosslinking CMC and chitosan and to incorporate them with iron oxide (Fe_3_O_4_) to remove Cr (VI) and Pb (II). Chitosan was crosslinked with CMC to improve the durability, strength, and functionality of CMC, along with the addition of Fe_3_O_4_ to create magnetite properties to facilitate the separation process from aqueous solution. The hydrogel beads (CMC-CS-Fe_3_O_4_) demonstrate a valuable characteristic, revealing a distinctive outcome compared to other research since they showed an increase in adsorption capacity after several cycles of regeneration, indicating the effectiveness of this polymer. Effective and reliable treatment for the removal of toxic and high mobility metals, such as Cr (VI) [34] and Pb (II), is necessary to reduce the risk of contamination. It is expected that this research will be able to provide insight into and alternatives for the usage of natural adsorbent, such as carboxymethyl cellulose and chitosan, in wastewater treatment to promote low toxicity, functional effects, and reasonable cost for a sustainable environment.

## 2. Results and Discussion

### 2.1. Crosslinking of CMC–Chitosan

**Figure 1:**
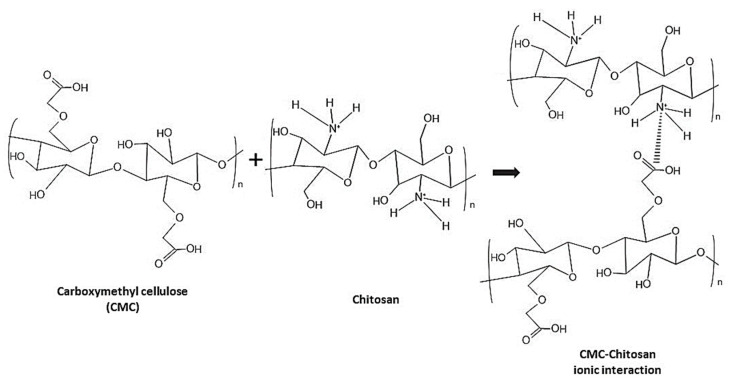
Proposed reaction mechanism of CMC and chitosan interaction.

When CMC and chitosan were mixed together, they formed a viscous mixture and agglomerating substance, which is likely impossible to be uniformly mixed and to pass through a syringe with a small inner diameter of 0.4 mm. During ionic crosslinking between carboxyl and amino groups in CMC and chitosan (Figure 1), the mixture produced a thick consistency because some of the carboxyl groups in CMC and hydroxyl groups in chitosan form strong hydrogen bonds, making them unsuitable for retaining water. The addition of H^+^ would break some of the hydrogen bond formed between CMC and chitosan and therefore be able to reduce the viscosity and improve the solubility between these polymers [22]. Hence, the pH of the mixture was decreased to around pH 1–2 using hydrochloric acid (HCl).

### 2.2. Swelling Capacity of CMC-CS-Fe_3_O_4_ Hydrogel Beads

**Figure 2:**
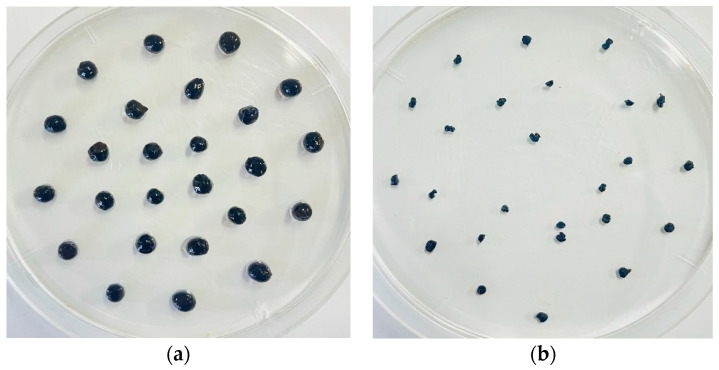
CMC-CS-Fe_3_O_4_ hydrogel beads. (**a**) Wet CMC-CS-Fe_3_O_4_ hydrogel beads; (**b**) CMC-CS-Fe_3_O_4_ hydrogel beads upon drying.

Figure 2 shows the hydrogel beads forming prior to drying and after 24 h of drying at 60 ℃. In swelling testing, the hydrogel beads showed moderate and great swelling percentages when immersed in deionized water for 24 h, as shown in Table 1. The R1 sample showed an average swelling ratio of 510% which is lower than that of R2 (840%), while R3 exhibited the highest swelling percentage, which was 2400%. The swelling ratio of these hydrogel beads may also indirectly indicate the water absorbency of these materials [35]. It can be observed here that, as the ratio of CMC increases, the swelling capacity of the hydrogel beads also increases because the CMC content contributes largely to the uptake and retention of water, as its structure is composed of a many functional carboxyl (–COO) and hydroxyl (–OH) groups [36]. Unlike CMC, chitosan generally does not contribute much to the swelling properties due to its NH_2_ content; instead, it is beneficial for holding the structure of the hydrogel beads. Hence, R3 hydrogel beads with the greatest swelling percentages is basically resulted from a balanced degree of ionic crosslinking between CMC and chitosan with fewer composition of amino groups [37], as too much crosslinking with NH_2_ will reduce the swelling capacity.

### 2.3. FTIR Characterization of CMC-Chitosan Hydrogel Beads

**Figure 3:**
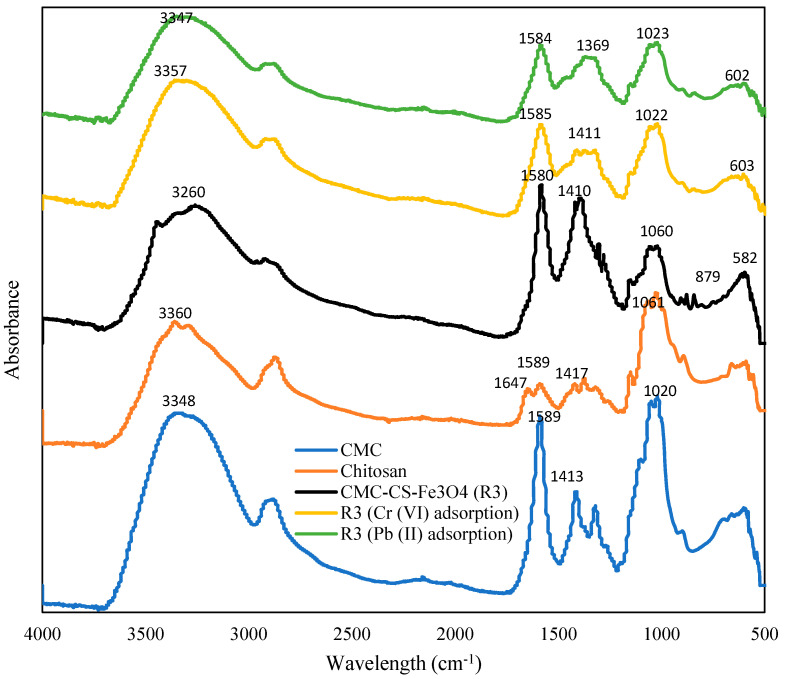
FTIR spectra of CMC, chitosan, and CMC-CS-Fe_3_O_4_ before and after adsorption of Cr (VI) and Pb (II).

CMC-CS-Fe_3_O_4_ hydrogel beads synthesized from CMC and chitosan (before and after adsorption of Cr (VI) and Pb (II)) were analyzed using Fourier-transform infrared spectroscopy (FTIR) to identify the crosslinking interactions between functional groups in the hydrogel. The synthesis of CMC-CS-Fe_3_O_4_ hydrogel beads demonstrated the absorption bands between CMC and CS. The FTIR spectrum of CMC in Figure 3 illustrates a broad and high intensity peak between 3600 cm^–1^ and 3200 cm^–1^, indicating –OH bending [25,38], while a relatively high and sharp peak at 1589 cm^−1^ characterizes the COOH groups [39]. In comparison, in chitosan, there are two distinctive peaks, which correspond to the amide groups, specifically amide-I band and amide-II band at 1647 cm^−1^ and 1589 cm^−1^, respectively [22]. On the other hand, the broad peak between 3600 cm^−1^ and 3250 cm^−1^ refers to the stretching vibrations of the –H and –NH_2_ groups [40].

The peaks at 879 cm^−1^ and 1060 cm^−1^ in the R3 spectra were found to be the saccharide structure and ether (–CO) functional groups [20,24], while the high intensity peak at 1410 cm^−1^ in R3 refers to the C–N stretching vibration [41]. The intense peak formed at 582 cm^−1^ in the R3 sample could be ascribed to the Fe–O bond, indicating the adsorption peak for Fe_3_O_4_ [42]. In the spectrum of the R3 sample, the moderate intensity peak formed at 1580 cm^−1^ could possibly be due to the ionic interaction between the carboxyl group of CMC and the amine functional group in chitosan [39]. The outcome shows that CMC-CS hydrogel polymers were formed through the ionic and electrostatic interactions between CMC and chitosan [37,43]. Furthermore, the peak of the amide band at 1647 cm^−1^ in chitosan became significantly reduced or almost vanished for the crosslinking of CMC-CS-Fe_3_O_4_ hydrogel beads [44,45], probably because some of the crosslinking interactions and hydrogen bonds formed between CMC and chitosan collapsed due to the addition of HCl. Therefore, it can be observed that CMC-CS-Fe_3_O_4_ hydrogel beads demonstrate absorption peaks from both CMC and chitosan.

At 1580 cm^−1^, the COO^−^ peak intensity of hydrogel beads (R3) remained high, as citrate and hydrogel mixtures formed crosslinking, as well as weak interfaces with Fe_3_O_4_. The crosslinked hydrogel beads showed a combination of different characteristic bands, which could be attributed by CMC, chitosan, and iron (Fe). Peaks at 3260 cm^−1^, 1580 cm^−1^, 1410 cm^−1^ and 1060 cm^−1^ in R3 showed reduced intensity peaks after adsorption of Cr (VI) and Pb (II), suggesting complex interactions between Cr^6+^ and Pb^2+^ and CMC-CS-Fe_3_O_4_ hydrogel beads. The changes and variable intensity of the peaks in the FTIR spectra of CMC, CS, and CMC-CS-Fe_3_O_4_ hydrogel beads (before and after adsorption) clearly signified the interaction between these polymers and metals.

### 2.4. Morphological Analysis by SEM

**Figure 4:**
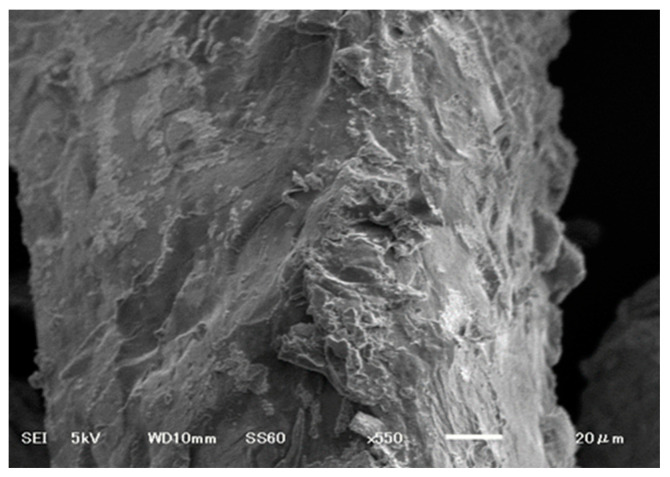
SEM micrograph of CMC-CS-Fe_3_O_4_.

Figure 4 shows the SEM images for CMC-CS-Fe_3_O_4_ hydrogel beads with a CMC:CS ratio of 3:1. The SEM images illustrate a rough and irregular morphology. The crosslinking among CMC, chitosan, and Fe_3_O_4_ shows a slightly rough structure, indicating balanced crosslinking interaction formed between the polymers. The morphology formed was attributable to ionic and electrostatic bonding between carboxyl and amine functional groups, which enabled the rough surfaces to contribute to the great absorbency and swelling capability [46].

### 2.5. Batch Adsorption of Cr (VI) and Pb (II) by Using CMC-CS-Fe_3_O_4_ Hydrogel Beads

#### 2.5.1. Effect of CMC-Chitosan Ratio

**Figure 5:**
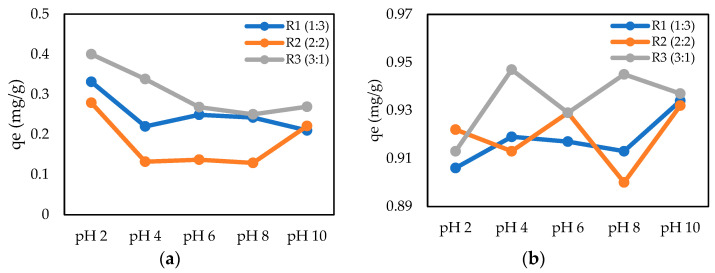
Ratio of CMC to CS at different pH values. (**a**) Adsorption of Cr (VI); (**b**) adsorption of Pb (II) (CMC-Cs-Fe_3_O_4_: 0.1 g, metal conc: 5 mg/L, volume: 20 mL, time: 60 min, and temperature: 30 °C).

**Figure 6:**
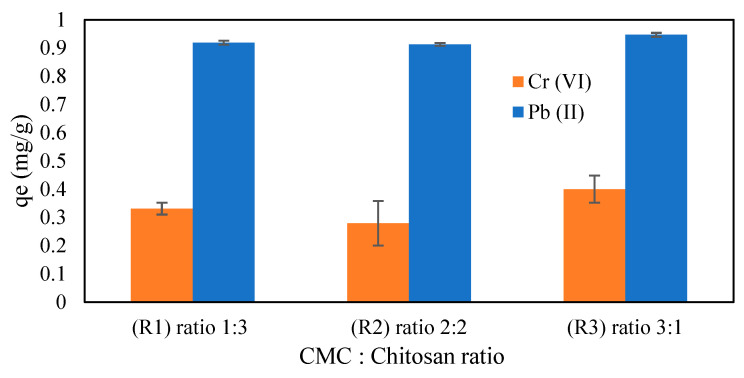
Ratios of CMC to CS for CMC-Cs-Fe_3_O_4_ at a pH of 2 [Cr (VI)] and a pH of 4 [Pb (II)] (CMC-Cs-Fe_3_O_4_: 0.1 g, metal concentration: 5 mg/L, volume: 20 mL, time: 60 min, and temperature: 30 °C).

In this study, different ratios of CMC to CS were impregnated with magnetic iron oxide (Fe_3_O_4_) solution and tested in several pH ranges for the adsorption of Cr (VI) and Pb (II). The effects of different ratios of CMC and chitosan in this experiment were studied to have a better understanding of the adsorption capacity. According to the results shown in Figure 5, the ratios of CMC to chitosan influenced the structure of the hydrogel beads and the adsorption capacity of this hydrogel for heavy metals at certain pH levels. During the synthesis of CMC–chitosan, the amounts of CMC and chitosan used varied based on certain ratios. The ratios of 1:3, 2:2, and 3:1 were used in this experiment for CMC and chitosan values. In the synthesis of these hydrogel beads, the total weights of CMC and chitosan used were kept constant at 0.5 g in 20 mL of 1% acetic acid except that the weight ratio of these two adsorbents was modified. Figure 5 depicts the results for the effect of the CMC–chitosan ratio at different pH levels on Cr (VI) and Pb (II) adsorption. According to the graph shown, the R3 sample with a CMC:chitosan ratio of 3:1 obtained the highest adsorption capability for Cr (VI) and Pb (II) at pH levels of 2 and 4, respectively. As mentioned before, CMC content contributed to the water retention and adsorption ability owing to its structure, which is composed of many functional carboxyl (–COO) and hydroxyl (–OH) groups [36]. Therefore, the CMC content allows for the formation of balanced crosslinks with the amino group in the chitosan structure. The lower adsorption capacity observed in the R1 and R2 samples may be due to the increasing ionic bonding between CMC and chitosan, which restricts the binding sites available for adsorbing metal ions. Therefore, the highest adsorption capacity observed in the R3 sample with a CMC:chitosan ratio of 3:1, as shown in Figure 6, was used for further analysis to study the effects of different parameters on the adsorption of Cr (VI) and Pb (II).

#### 2.5.2. Influence of pH on Cr (VI) and Pb (II) Adsorption

The pH of metal solutions is one of the most important factors strongly influencing the interactions between metals and the adsorbent [47]. Different pH levels will affect the surface’s interaction between the adsorbent and the adsorbate. Therefore, evaluations of the optimum pH conditions for the adsorption process were conducted to understand the mechanisms. For this experiment, 5 mg/L of Cr (VI) and Pb (II) solutions with different pH ranges from 2 to 10 were tested with a constant weight of CMC-CS-Fe_3_O_4_ (0.10 g) for 60 min.

**Figure 7:**
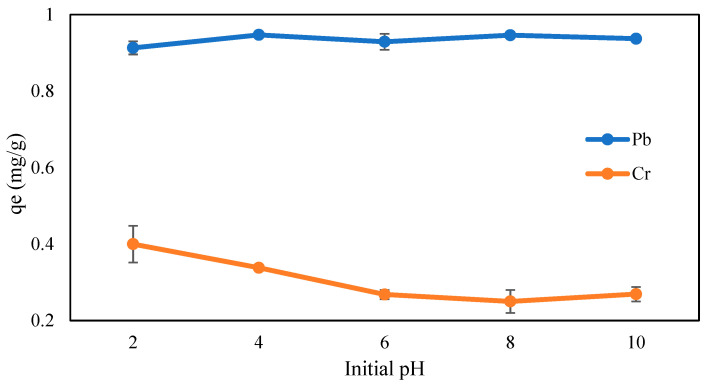
Influence of initial pH on Cr (VI) and Pb (II) adsorption (CMC-Cs-Fe_3_O_4_: 0.1 g, metal concentration: 5mg/L, volume: 20 mL, time: 60 min, and temperature: 30 °C).

Based on the results presented in Figure 7. The adsorption capacity of Cr (VI) decreased from 0.40 mg/g to 0.25 mg/g as the pH increased from 2 to 8, and the adsorption slightly rose to 0.269 mg/g at a pH of 10. From the results, it can be clearly observed that the maximum removal and adsorption capacity of Cr (VI) was obtained at a pH of 2, whereas for the adsorption capacity of Pb (II), the adsorption amount increased from 0.913 mg/g (pH 2) to 0.947 mg/g at a pH of 4. At a pH of 6, the adsorption began to decline to 0.929 mg/g and suddenly increased to 0.946 mg/g at a pH of 8 and finally decreased to 0.937 mg/g at a pH of 10. It can be seen that the differences in adsorption capacity with respect to Pb (II) are negligible over the pH ranges since there are no significant changes observed; however, a pH of 4 was selected as the experimental condition for further analysis of Pb (II) adsorption.

**Figure 8:**
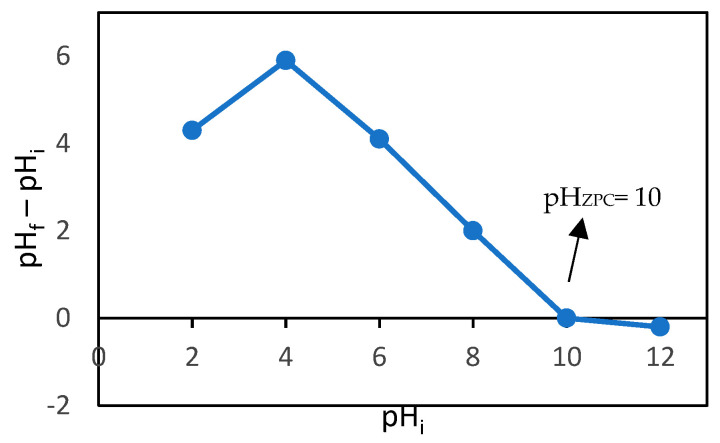
Point of zero charge of CMC-CS-Fe_3_O_4_.

Cr (VI) at a pH of 2 exists in the form of Cr_2_O_7_^2−^ and HCrO_4_^−^, which are negative ions [48]. The pH_zpc_ of the CMC-CS-Fe_3_O_4_ hydrogel beads was determined to obtain information regarding the pH value at which the charge of adsorbent’s surface is equal to zero. Determination of the zero-point charge is important to properly understand the adsorbent’s surface charge at a certain pH. Based on Figure 8, the pH_ZPC_ of CMC-CS-Fe_3_O_4_ hydrogel beads was discovered to be 10. At a pH less than 10, the surface of adsorbent becomes positively charged and more prone to protonation [49]. Hence, the negative ions of chromium (Cr_2_O_7_^2−^/HCrO_4_^−^) existing at a pH of 2 favor the interaction forces with positively charged amino groups in CMC-CS-Fe_3_O_4_ and are capable of removing the heavy metal from aqueous solutions. The surface of the adsorbent, which is positively charged, is able to form electrostatic interactions with the Cr (VI) ions [44] and therefore results in higher adsorption capacity and a greater removal percentage of chromium.

As for Pb (II), it normally presents in the form of positive ions (Pb^2+^) at pH 4 [50]. At a low pH, the acidic functional group on the CMC-CS-Fe_3_O_4_ adsorbent will have a repulsion charge with cationic Pb (II), resulting in low adsorption capacity. It can be observed that the adsorption capacity was low at a pH of 2 and rapidly increased as the pH increased from 2 to 4. Additionally, at a lower pH, there are many hydrogen ions available, and they will compete with Pb^2+^ [51] to bind to the active site (COO^−^ group) of the adsorbent. As a result, the presence of H^+^, which dominates at a low pH, will prevent the adsorbent from interacting with cationic metals. Therefore, CMC-CS-Fe_3_O_4_ hydrogel beads were able to adsorb Pb^2+^ and become stable at a slightly higher pH (pH 4) due to the reduced concentration of hydrogen ions [52]. A similar trend was observed by Li et al. [50], in which a composite adsorbent containing Fe_3_O_4_ was found to greatly adsorb Pb (II) at a pH of 4. Based on the results presented in Figure 7, the best adsorption capacities of Cr (VI) and Pb (II) were obtained at pH values of 2 and 4, respectively.

#### 2.5.3. Influence of Initial Concentration

**Figure 9:**
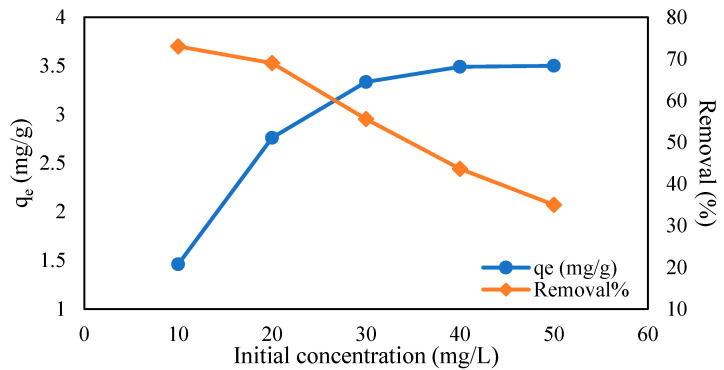
Effect of initial concentration on Cr (VI) adsorption (CMC-Cs-Fe_3_O_4_: 0.1 g, pH: 2, volume: 20 mL, time: 1800 min, and temperature: 30 ℃).

**Figure 10:**
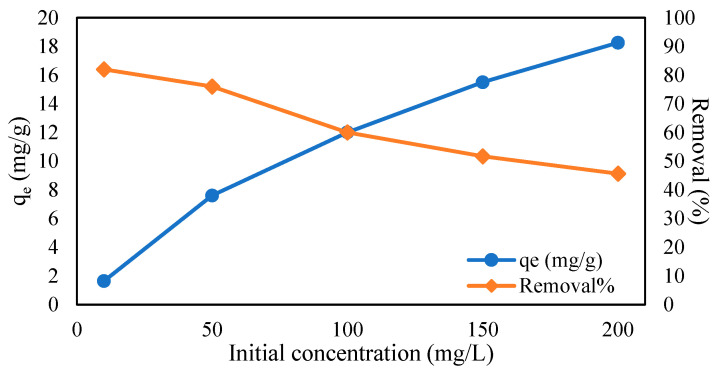
Effect of initial concentration on Pb (II) adsorption (CMC-Cs-Fe_3_O_4_: 0.1 g, pH: 2, volume: 20 mL, time: 1800 min, and temperature: 30 ℃).

The effects of initial concentrations of metal solutions were studied to have a better understanding of the adsorption capacity. The initial concentrations of Cr (VI) solutions were tested at several ranges of 10 to 50 mg/L, and adsorbate solutions of Pb (II) were adjusted from 10 to 200 mg/L, with parameters such as pH, time, and the adsorbent’s weight kept constant for this experiment. According to the graph in Figure 9, the adsorption capacity of Cr (VI) was discovered to increase as the initial concentration increased. This trend applied the same to the Pb (II) adsorption capacity by R3 hydrogel beads in Figure 10. The adsorption capacity for both Cr (VI) and Pb (II) increased from 1.46 to 3.5 mg/g and 1.64 to 18.26 mg/g due to the increased availability of metal ions, allowing more metal ions to bind to the adsorbent [51,53,54]. The greater availability of metals ions tends to occupy the adsorbent sites, leading to greater driving forces [53] and therefore increased adsorption capacity. However, the removal percentages seemed to decrease as the initial concentrations of adsorbate increased. Similar trends have been validated by many researchers, such as Karthik et al. [55], as the adsorbent surface becomes unavailable due to the increased saturation of the sorption site. Furthermore, it can be observed that the adsorption value of magnetite hydrogel beads for Cr (VI) was much lower than that for Pb (II), which may be due to certain factors, such as the metal ionic radius, electronegativity, or charge density. Pb^2+^ is known to have a smaller hydrated radius than Cr^6+^ (Pb^2+^ = 0.401 nm, Cr^6+^ = 0.461) [56,57]. The smaller size of Pb^2+^ enables stronger attraction to the adsorbent’s surface compared to Cr^6+^.

#### 2.5.4. Adsorption Isotherms

The initial metal concentration is certainly a significant parameter that affects the heavy metal adsorption capacity [58]. In this research, the adsorption isotherms are presented to represent the correlation between the number of metal ions adsorbed into the adsorbent and the concentrations of metal solutions after reaching equilibrium.

In this study, 0.1 g of CMC-CS-Fe_3_O_4_ beads were mixed into 25 mL of Cr (VI) and Pb (II) solutions at a constant temperature of 30 ℃ for 1800 min. The adsorption capacity of CMC-CS-Fe_3_O_4_ hydrogel beads with respect to Cr (VI) and Pb (II) was reported to increase as the initial concentration increased from 10 to 50 mg/L and 10–200 mg/L, respectively. The adsorption isotherm of this study is characterized using Langmuir and Freundlich equations. Both equation models are presented in Table 2. 

**Figure 11:**
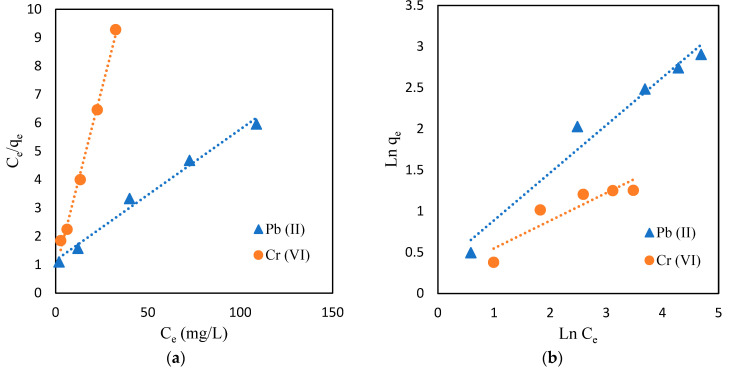
Adsorption isotherm studies. (**a**) Langmuir isotherm; (**b**) Freundlich isotherm (CMC-Cs-Fe_3_O_4_: 0.1 g, pH Cr (VI): 2, pH Pb (II): 4, volume: 20 mL, time: 1800 min, and temperature: 30 °C).

The results for isotherm model curves are illustrated in Figure 11. Between the two isotherm models, the Langmuir model was found to be the better-fitted models for Cr (VI) and Pb (II) removal in this study, as it obtained a higher R^2^ compared to Freundlich. It is essential to evaluate the R^2^ value to determine the suitability and to confirm the adsorption isotherm models. Table 3 shows the data for isotherm constants and the resulting fitting correlation coefficients (R^2^) for both models, Langmuir and Freundlich. This outcome shows that the experiments followed the Langmuir isotherm, which implements monolayer adsorption, instead of multilayer adsorption and heterogenous systems (Freundlich model) [59]. Therefore, according to the Langmuir model, there will be no further adsorption processes occurring once the binding sites have been occupied, and it is a reversible chemical reaction, in which adsorption and desorption are equal at equilibrium [60]. Moreover, in this study, the Langmuir equilibrium parameters R_L_ shows a value <1 for both Cr (VI) and Pb (II), indicating that this type of adsorption is favorable.

#### 2.5.5. Influence of Contact Time, and Adsorption Kinetics Studies

**Figure 12:**
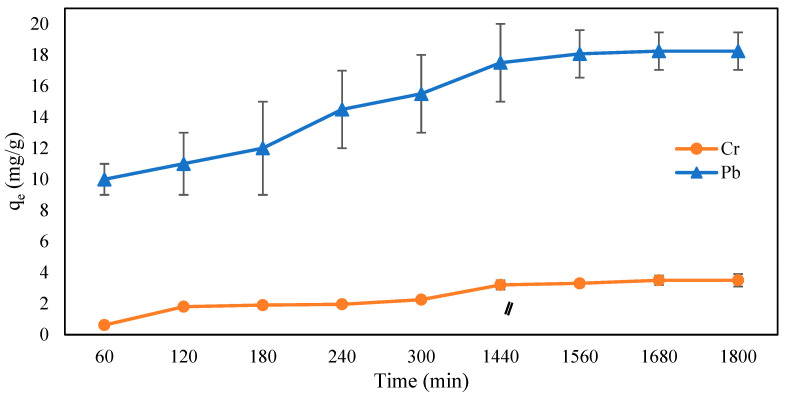
Influence of contact time on Cr (VI) and Pb (II) adsorption (CMC-Cs-Fe_3_O_4_ dosage: 0.1 g, volume: 20 mL, concentration: 50 mg/L at pH 2 for Cr (VI), 200 mg/L at pH 4 for Pb (II), and temperature: 30 °C).

In an attempt to properly understand the influences of time and adsorption kinetics of Cr (VI) and Pb (II) by CMC-CS-Fe_3_O_4_ hydrogel beads, an adsorption experiment was conducted for certain period of times (60–1800 min) to investigate the effect of contact time on adsorption capacity. As shown Figure 12, the adsorption capacity increased at the initial stage from 60 to 300 min and started to slow until reaching equilibrium at 1680 min. The rapid adsorption in the initial stage was attributed to the availability of functional groups of CMC-CS-Fe_3_O_4,_ leading to interaction between the Cr (VI) ions and Pb (II) ions with the CMC-CS-Fe_3_O_4_ surfaces. The adsorption process gradually slowed as the metal ions occupied the active site of CMC-CS-Fe_3_O_4_ until it finally reached equilibrium adsorption capacity of 3.5 and 18.26 mg/g for Cr (VI) and Pb (II), respectively.

Contact time is an important parameter in the adsorption process and can specify the information related to adsorption kinetics of an absorbate at a constant dosage of adsorbent. Therefore, adsorption kinetics, such as pseudo-first order (PFO) and pseudo-second order (PSO), were employed in the experimental data to obtain the information related to the mechanism of the adsorption process.

In adsorption kinetics, the pseudo-first order generally represents the number of metal ions (adsorbate) adsorbed into the adsorbent, and the rate of adsorption depends on the concentration of adsorbate [61], whereas the pseudo-second order model is controlled by chemisorption, and it depends on the adsorption capacity. The linear equations of the pseudo-first order (Equation (1)) and pseudo-second order (Equation (2)) are listed as follows:

(1) $$\log\left(q_{e}-q_{t}\right)=\log q_{e}-(\frac{k_{1}t}{2.303})$$

(2) $$\frac{t}{q_{t}}=\frac{1}{k_{2}q_{e}^{2}}+\frac{1}{q_{e}}$$

where k_1_ is the rate constant for the pseudo-first order (min^−1^); k_2_ is the rate constant for the pseudo-second order (g mg^−1^ min^−1^); q_e_ refers to the amount of metals adsorbed (mg/g); and t is the time.

**Figure 13:**
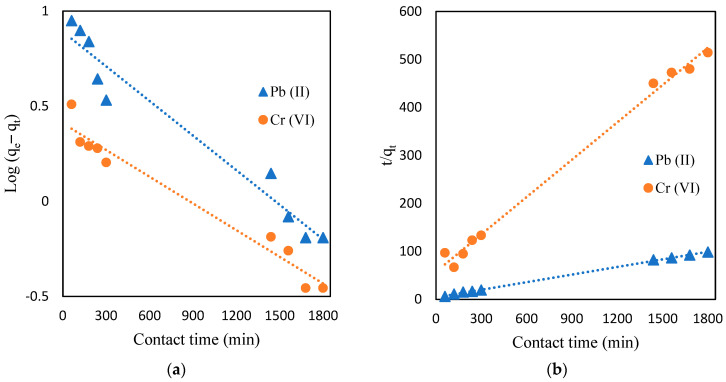
Adsorption kinetic studies. (**a**) Pseudo-first order; (**b**) pseudo-second order (CMC-Cs-Fe_3_O_4_ dosage: 0.1 g, volume: 20 mL, concentration: 50 mg/L at pH 2 for Cr (VI), 200 mg/L at pH 4 for Pb (II), and temperature: 30 °C).

The results for adsorption kinetics are shown in Table 3. Based on Figure 13, the pseudo-second order provided the best correlation of our experimental data for adsorption kinetics of both Cr (VI) and Pb (II). This outcome signifies that the adsorption process is controlled by chemisorption since it depends on the adsorption capacity instead of the adsorbate’s concentration [62]. The adsorption process was attributed to the functional groups and active sites present in the CMC-CS-Fe_3_O_4_ hydrogel beads [63,64]. The best fit of kinetic models can be determined by calculating the predicted amount of metal adsorbed (q_e_) and the correlation coefficient value (R^2^). It can be clearly observed from Table 4 that the values of the correlation coefficient (R^2^) for Cr (VI) and Pb (II) were relatively higher in the PSO model. Moreover, the PSO model can provide information regarding the theoretical value for equilibrium adsorption capacity through calculation from the experimental data [65].

Table 4 shows the comparison of adsorption capacities determined by previous research using various types of adsorbents synthesized from chitosan and carboxymethylcellulose polymer.

### 2.6. Desorption Studies

To investigate the regeneration of CMC-CS-Fe_3_O_4_ hydrogel beads as adsorbent agents in heavy metal removal, a desorption study was carried out to properly understand the characteristics and potential of these hydrogel beads. The desorption process was conducted by mixing the adsorbed hydrogel beads with different solvents for 5 h at 30 ℃ to determine the types of solvents with the best desorption performance. The types of eluents used were 0.1 M HCl, 0.3 M NaOH, and 0.2 M H_2_SO_4_ for Cr (VI) [34,70,71], while the eluents used for Pb (II) desorption were 0.3 M NaOH, 0.2 M H_2_SO_4_, and 0.01 M Na2·EDTA [41,51,71]. Based on Table 5, the usage of 0.3 M NaOH and 0.01 M Na2·EDTA showed the greatest performance for Cr (VI) and Pb (II) desorption, respectively. In this study, the desorption mechanism of Cr (VI) might be influenced by ion exchange interaction [63,64], whereas in the case of Pb (II) desorption, Na2·EDTA had higher complexing ability than the hydrogel beads; therefore, Pb^2+^ was complexed by ethylenediaminetetraacetic acid disodium. Acidic solutions, such as 0.3 M HCl, have negative effects on Cr (VI) desorption and result in the decomposition of the structure of CMC-CS-Fe_3_O_4_ hydrogel beads, whereas diluted acid solutions, such as 0.2 M H_2_SO_4_, have a slight impact for the recovery of adsorbed Cr (VI) and Pb (II).

**Figure 14:**
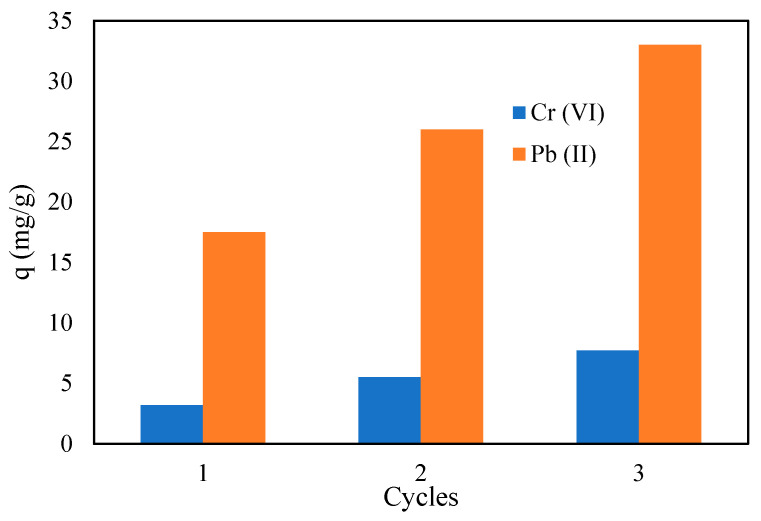
Adsorption capacity of CMC-Cs-Fe_3_O_4_ after several regeneration cycles. (CMC-Cs-Fe_3_O_4_ dosage: 0.1 g, volume: 20 mL, concentration: 50 mg/L at pH 2 for Cr (VI), 200 mg/L at pH 4 for Pb (II), time: 1440 min, and temperature: 30 ℃).

For the regeneration of CMC-CS-Fe_3_O_4_, the second cycles of adsorption and desorption processes showed an unexpected increase in adsorption capacity for both heavy metals compared to the first one, as shown in Figure 14. A similar trend was observed after the third cycle, in which the adsorption capacity increased to 7.7 mg/g and 33.0 mg/g for Cr (VI) and Pb (II), respectively. The sudden increase in adsorption capacity compared to the first cycle may be explained by the case in which the metal ions entrapped inside the beads create more spaces and provide access to metal ions to bind with active sites inside the beads during the subsequent adsorption cycles [72]. In another case, it could be due to the thinning of the outer layer of the beads, leading to greater exposure to active sites. This information corresponds to the FTIR data obtained from CMC-CS-Fe_3_O_4_ beads after third desorption cycles, which showed a great increase in the intensity of NH_2_ bending and COOH groups. Therefore, this finding indicates that the hydrogel beads become more stable and effective after several regenerations.

## 3. Conclusions

The magnetite carboxymethyl cellulose–chitosan bio-adsorbent formation through ion crosslinking was synthesized and examined for its potential to adsorb heavy metals, namely Cr (VI) and Pb (II), from aqueous solution. The experimental data showed that CMC-CS-Fe_3_O_4_ hydrogel beads have reliable adsorption capacity due to the presence of active sites and functional groups, such as carboxyl and amino groups. It can be reported that the optimal pH for Cr (VI) and Pb (II) adsorption was at 2 and 4, respectively. The maximum adsorption capacities obtained for both Cr (VI) and Pb (II) were 3.5 mg/g and 18.26 mg/g, respectively, after contact time of 28 h. The adsorption isotherms were described by Langmuir, where the value of the equilibrium parameter R_L_ is favorable (0 < R_L_ < 1). For Cr (VI) and Pb (II), the adsorption kinetics were best fitted to pseudo-second order kinetic models, which explained that the specific surface area and chemical sorption highly influence the adsorption process. The experimental data show that desorption of Cr (VI) and Pb (II) from the hydrogel beads was achieved at 70.43% and 83.85%, respectively, using 0.3 M NaOH and 0.01 M Na2·EDTA. Moreover, this polymer showed an unexpected increase in adsorption capacity after several cycles of the adsorption–desorption process. Overall, the CMC-CS-Fe_3_O_4_ hydrogel beads showed good adsorption ability, which can be easily separated and recovered from aqueous solution. Thus, the results and information about this research could provide insights into the potential and reliable use of this bio-polymer adsorbent for wastewater treatment in a sustainable environment.

## 4. Materials and Method

### 4.1. Chemicals and Reagents

Chitosan powder (molecular weight of 100,000 to 300,000 Da) was supplied by Acros Organics (Shanghai, China). Carboxymethyl cellulose (CMC) powder was obtained from Katayama Chemical. Iron (III) chloride (FeCl_3_, 38%) was purchased from Hayashi Pure Chemical Ind. (Osaka, Japan), and iron (II) sulfate (FeSO_4_, 99%) was obtained from Kanto Chemical Co. (Tokyo, Japan). All the chemicals and reagents used in this study were of chemical grade.

### 4.2. Preparation and Crosslinking of CMC-CS-Fe_3_O_4_ Hydrogel Beads

A volume of 20 mL of 2% acetic acid solution was prepared and stirred at 2000 rpm. For the R1 sample, 0.375 g of chitosan and 0.125 g of CMC were weighed and added slowly to the acetic acid solution. The mixture was stirred for about 30 min. The pH of the mixture was then adjusted to around pH 1–2 by adding 1 M HCl to improve the consistency and solubility of the CMC–chitosan mixture.

Magnetic hydrogel beads were prepared by adding 1 M of FeCl_3_ and 0.5 M of FeSO_4_ to the CMC–chitosan mixture and stirring it for 30 min. After proper mixing, the mixture was added dropwise into an alkaline solution containing 1 M NaOH and 0.1 M sodium tricitrate to create uniform and round beads using a 10-mL syringe. Sodium tricitrate was incorporated into the alkaline NaOH solution to increase the stability of the hydrogel beads. The formed beads were allowed to set for about 24 h in the alkaline solution. Last, 0.1 M HCl and deionized water were used to wash the beads thoroughly and remove the residual alkali before drying them in an oven at 60 ℃ for about 24 h. The above procedures were repeated for other ratios of CMC to chitosan (R2 and R3), as shown in Table 6 below.

### 4.3. Swelling Capacity of CMC-CS-Fe_3_O_4_ Hydrogel Beads

The swelling capacity of CMC-CS-Fe_3_O_4_ with different CMC:Cs ratios was studied by soaking 0.1 g of CMC-CS-Fe_3_O_4_ hydrogel beads in 100 mL of distilled water for 24 h. The swollen beads were placed on blotting paper to remove excess water and were weighed to calculate the swelling percentage using Equation (3):

(3) $$Swelling\;percentage=\frac{m_{f}-m_{i}}{m_{i}}\times 100$$

where $m_{i}$ and $m_{f}$ are the initial and final weights (g) of CMC-CS-Fe_3_O_4_ hydrogel beads, respectively.

### 4.4. Characterization of CMC-CS-Fe_3_O_4_ Hydrogel Beads

The spectra of FT-IR for CMC, chitosan, and CMC-CS-Fe_3_O_4_ before and after Cr (VI) and Pb (II) adsorption were examined using ATR-FTIR (Thermo Scientific Nicolet iS10, Waltham, MA, USA) to determine the functional groups of the hydrogel beads. The structure and morphology of CMC-CS-Fe_3_O_4_ hydrogel beads were analyzed by SEM (JIED-2300, Shimadzu, Kyoto, Japan). For the determination of the zero-point charge (pH_ZPC_) of CMC-CS-Fe_3_O_4_ hydrogel beads, 50 mL of 0.01 NaCl solutions with different initial pH values (2–12) were used as electrolyte solutions. Next, 0.1 g of adsorbent was added to the NaCl solution and shaken for 24 h. The final pH of the solutions were measured, and the pH difference obtained was plotted against the initial pH. The pH at which the graph intersects zero or the electric charge density of the adsorbent is zero was taken as pH_ZPC_.

### 4.5. Batch Adsorption Experiment

To assess the influence of pH on the adsorption capability of CMC-CS-Fe_3_O_4_ hydrogel beads for Cr (VI) and Pb (II), 5 mg/L of Cr (VI) and Pb (II) solutions were prepared for adsorption experiments. A mass of 0.1 g of CMC-CS-Fe_3_O_4_ hydrogel beads was introduced into 20 mL of the Cr (VI) and Pb (II) solutions with different pH ranges (2, 4, 6, 8, 10) and shaken at 100 rpm using a mix rotor (VMRC-5) for 60 min at 30 ℃. The hydrogel beads were separated from the solution using an external magnet, and the final concentrations of the Cr (VI) and Pb (II) solutions were analyzed using a heavy metal test kit and spectrophotometer (Kyoritsu Chemical-Check Lab., Corp, Yokohama, Japan) to calculate the adsorption amounts ($q_{e}$) and removal percentage by Equations (4) and (5):

(4) $$q_{e}=\frac{\left(C_{i}-C_{e}\right)}{M}\times V$$

(5) $$\%removal=\frac{\left(C_{i}-C_{e}\right)}{C_{i}}\times 100$$

where $C_{i}$ and $C_{e}$ are the concentrations of the Cr (VI) and Pb (II) solutions (mg/L) during the initial and final concentrations, respectively. M is the weight of the adsorbent (g), and V is the volume of the metal solution in liters (L).

Further analysis of the influences of CMC:CS ratio, initial adsorbate [Cr (VI) and Pb (II)] concentrations, and contact time was conducted. To evaluate the adsorption isotherm, two isotherm models, which were the Langmuir and Freundlich models, were used to analyze the experimental equilibrium data between the adsorbate and the adsorbent. Furthermore, the adsorption kinetics of this material were also evaluated by employing pseudo-first order (PFO) and pseudo-second order (PSO) models. The PFO model is commonly controlled by physisorption, as it involves a diffusion mechanism process, whereas PSO is controlled by chemisorption. Sometimes, it can be assumed that, in a PSO model, the rate of adsorption (attachment to the adsorbent’s surface) increases when the rate of sorption decreases. All the experiments were conducted in duplicate, and the experimental data were used for further analysis.

### 4.6. Desorption and Regeneration

In this experiment, different eluent solutions were employed to determine the best desorption capability for Cr (VI) and Pb (II) ions without degrading the structure and quality of the CMC-CS-Fe_3_O_4_ hydrogel beads. Several types of solutions used for desorption of Cr (VI) include 0.1 M HCl, 0.3 M NaOH, and 0.2 M H_2_SO_4_, whereas for Pb (II) ions desorption, 0.3 M NaOH, 0.2 M H_2_SO_4_, and 0.01 M Na·EDTA were used. First, 0.1 g of CMC-CS-Fe_3_O_4_ hydrogel beads were subjected to an adsorption process in 20 mL of Cr (VI) and Pb (II) solution with concentrations of 50 mg/L and 200 mg/L, respectively. After adsorption, the hydrogel beads were separated from the aqueous solution and dried in an oven before the desorption process. For desorption experiments, the dried hydrogel beads were introduced into 20 mL of the desorption eluents for 5 h at 30 ℃. Subsequently, the concentrations of Cr (VI) and Pb (II) ions were measured using a heavy metal test kit and spectrophotometer (Kyoritsu-lab). The percentages of desorbed metals were calculated using Equation (6):

(6) $$Desorption\%=\frac{Released\;metal\;concentration}{Initially\;adsorbed\;metal\;concentration}\times 100$$

## Figures and Tables

**Table 1:** Properties and swelling percentages of CMC-CS-Fe_3_O_4_ hydrogel beads.

Hydrogel Samples	(CMC:Chitosan) Ratio	Bead’s Shape upon Drying	Swelling Percentage (%)
R1	(1:3)	Able to retain round shape	510%
R2	(2:2)	Able to retain round shape	840%
R3	(3:1)	Round shape and sometimes flat	2400%

**Table 2:** Parameters of isotherm models for Cr (VI) and Pb (II) adsorption by CMC-CS-Fe_3_O_4_.

Adsorption Isotherm	Equation	Parameters	Cr (VI)	Pb (II)
Langmuir	$\frac{C_{e}}{q_{e}}=\frac{1}{q_{m}.K}+\frac{C_{e}}{q_{m}}$ $R_{L}=\frac{1}{\left(1+\left(K_{L}\times C_{i}\right)\right)}$	$q_{m}$ $K_{L}$ $R_{L}$ R^2^	3.926188 0.30628 0.03162 0.9940	21.69197 0.03970 0.20122 0.9866
Freundlich	$Ln\;q_{e}=Ln\;K_{f}+\frac{1}{n}Ln\;C_{e}$	$K_{f}$ 1/n R^2^	1.2355 0.3368 0.8268	1.364 0.5784 0.9681

Where C_e_ is the equilibrium concentration of the adsorbate (mg/L), while, q_e_ and q_m_ are the equilibrium adsorption capacity (mg/g) and maximum adsorption capacity (mg/g), respectively. K_L_ is the equilibrium (Langmuir) constant of adsorption (L/mg), whereas C_i_ is the initial concentration (mg/L). R_L_ is the equilibrium parameter, indicating the type of adsorption, where R_L_ = 0 is irreversible, R_L_ > 0 is favorable, R_L_ > 1 is unfavorable, and at R_L_ = 1, the adsorption is linear. Last, K_f_ is the Freundlich constant (mg/g).

**Table 3:** Parameters of kinetic models for Cr (VI) and Pb (II) adsorption by CMC-CS-Fe_3_O_4_ hydrogel beads.

Adsorption Kinetic Models	Parameters	Cr (VI)	Pb (II)
Pseudo-first order	$q_{e}$ $K_{1}$ *R* ^2^	2.57 0.00115 0.9623	7.77 0.00138 0.9549
Pseudo-second order	$q_{e}$ $K_{2}$ *R* ^2^	3.85 0.00117 0.994	18.94 0.00068 0.9993

**Table 4:** The comparison of other CMC/chitosan/Fe_3_O_4_-based adsorbents.

Adsorbent Types	Heavy Metals	Adsorption Capacity	Desorption %	References
PVA/ chitosan magnetic composite	Co (II)	14.39 mg/g	97.5	[66]
Chitosan/CMC bio-based composite	Cr (IV) Cu (II) Pb (II) Ni (II) Zn (II)	0.537 mg/g 2.104 mg/g 16.35 mg/g 0.421 mg/g 1.198 mg/g	-	[29]
PVA/CMC	Ag Ni (II) Cu (II) Zn (II)	8.40 mg/g 6.00 mg/g 5.50 mg/g 5.30 mg/g	-	[67]
CMC–ECH hydrogel beads	Cu (II) Ni (II) Pb (II)	6.49 mmol/g 4.06 mmol/g 5.15 mmol/g	-	[36]
Carboxymethyl cellulose hydrogel	Cu (II)	2.30 mg/g	89.0	[68]
Magnetic chitosan/cellulose microsphere	Cu (II) Pb (II) Cd (II)	88.2 mg/g 45.8 mg/g 61.1 mg/g	92.0 85.0 89.0	[69]

**Table 5:** Desorption studies for Cr (VI) and Pb (II).

Eluent Solvents	Heavy Metals	
	Desorption % Cr (VI)	Desorption % Pb (II)
0.3 M HCl	0.0	-
0.3 M NaOH	70.43	13.08
0.2 M H_2_SO_4_	12.17	19.23
0.01 M Na2·EDTA	-	83.85

**Table 6:** Weight ratio of CMC:chitosan mixture.

Hydrogel Samples	CMC:Chitosan Ratio
R1	1:3
R2	2:2
R3	3:1
